# Bioactive Natural Molecules and Traditional Herbal Medicine in the Treatment of Airways Diseases

**DOI:** 10.1155/2016/9872302

**Published:** 2016-10-03

**Authors:** Alexandre de Paula Rogerio, Troy Carlo, Sergio R. Ambrosio

**Affiliations:** ^1^Departamento de Clínica Medica, Laboratório de Imunofarmacologia Experimental (LIFE), Instituto de Ciências da Saúde, Universidade Federal do Triângulo Mineiro (UFTM), 38025-350 Uberaba, MG, Brazil; ^2^Pulmonary and Critical Care Medicine Division, Brigham and Women's Hospital, Boston, MA 02115, USA; ^3^Núcleo de Pesquisa em Ciências e Tecnologia, Universidade de Franca, 14404-600 Franca, SP, Brazil

The use of herbal medicines has been documented throughout recorded history including Assyrian clay tablets (2000 BC) and the Egyptian Ebers Papyrus (1550 BC) and in Ayurveda works from 900 BC. In addition, Hippocrates, Dioscorides, Galen, Paracelsus, and Arab scholars in Europe kept detailed notes regarding the use of medicinal plants. In the modern era, the research of natural bioactive molecules began with the isolation of morphine from opium latex in 1805 by Sertürner. A famous bioactive compound isolated from plants, salicylic acid, was isolated from willow bark in the 19th century. In 1900, Bayer patented a derivative of the compound to treat pain and inflammation and sold it under the brand name aspirin. Numerous other natural compounds have been discovered and used for the treatment of diseases.

Considerable progress has been made in the understanding of genetic and immunological factors that contribute to the development of airway disorders such as asthma, chronic obstructive pulmonary disease, and acute lung injury. Unfortunately, current therapies and treatments for airway disorders have not advanced dramatically despite our increased knowledge base. Traditional herbal medicine and bioactive natural products are being used as therapeutic substitutes or as complementary treatments to augment existing therapies. The natural compounds found in these may have the potential to form the basis of new drugs for the treatment of diseases. In this special issue, we took a particular interest in reviews and original papers that investigate the effects of bioactive natural molecules and traditional herbal medicines in clinical and preclinical (cellular or animal models) studies in prevention and treatment of airways diseases.

Allergic asthma is an airway inflammatory disorder coordinated by CD4^+^ T cells with Th2 phenotype. The main characteristics of asthma are airway hyperresponsiveness, eosinophilic inflammation, and hypersecretion of mucus. Typical asthma treatments include *β*_2_-agonists and inhaled or systemic corticosteroids. In addition, inhibitors of LT synthesis (such as zileuton, which directly inhibits 5-LO) or CysLT1 antagonists (such as montelukast, zafirlukast, and pranlukast) may also be used as complementary therapies to treat asthma, reducing the requirement for corticosteroids. Although the drugs described above have potent effects when used individually or in combination, they also have adverse side effects that limit their long-term use. Thus, it is necessary to develop new compounds with similar therapeutic potential and less adverse effects for the continuous treatment of airway diseases. In an ongoing search for bioactive plant-derived natural products, several groups, including some of this special issue, have successfully employed experimental methods to screen plant extracts and isolate compounds for pharmacological activity.

Ferulic acid (4-hydroxy-3-methoxycinnamic acid) is found in a wide array of plants, including fruits and vegetables, and in beverages such as coffee and beer. Ferulic acid demonstrates antioxidant and anti-inflammatory activities suggesting a therapeutic potential to treat several diseases such as arthritis and Alzheimer's disease.

Using a classical allergic airway inflammation model in mice (induced by ovalbumin) C.-C. Lee et al. demonstrated ferulic acid reduced most phenotypes associated with asthma including IgE and IgG1 production, airway hyperactivity, airway inflammation, chemokine (eotaxin), and cytokines (IL-4, IL-5, IL-13, and TNF-*α*) amounts in bronchoalveolar lavages. In addition, ferulic acid increased IFN-*γ* (an inhibitor of Th2 immune response) and IL-12 (inductor of differentiation of naive T cells toward the Th1 phenotype) production as well as reduced proinflammatory cytokines (TNF-*α*, IL-1*β*, and IL-6) production by lipopolysaccharide-stimulated bone-marrow-derived dendritic cells. This effect was associated with the ability of ferulic acid polarize Th1 cells (increasing IFN-*γ*) increasing expression level of Notch ligand Delta-like 4 (Dll4) and reducing the Jagged1 mRNA synthesis by LPS-stimulated dendritic cells. In addition, expression of MHC class II and CD40 molecules during dendritic cells maturation was increased. In a mixed lymphocyte reaction assay, ferulic acid induces proliferation of T cells with an associated increase of IFN-*γ* and reduction of IL-5 suggesting that ferulic acid possesses an antiallergic effect by restoring Th1/Th2 imbalance by modulating dendritic cells function.

*Elaeagnus pungens* leaves, a flowering plant from the Elaeagnaceae family, are used in traditional Chinese medicine to treat severe asthma, cough, bronchitis, or other respiratory disorders. Y. Ge et al. evaluated the role of aqueous extracts of* E. lanceolata*,* E. henryi*, and* E. pungens* to alleviate symptoms in an asthma model (induced by mixture spray of histamine and acetylcholine chloride) in guinea pigs, a tussive model (induced by exposure to aqueous ammonia) in mice, and an expectorant assay (measurement of the concentration of phenol red photometrically in whole trachea dissected from animals) in mice. All extracts demonstrated antiasthmatic, antitussive, and expectorant activities. These effects were associated with presence of flavonoids compounds in the leaves.

People with antigen-specific IgE antibodies and mast cells dependent allergic rhinitis can experience early phase symptoms (such as sneezing and rhinorrhea) within minutes of exposure to the associated allergens. However, such individuals also typically develop the nasal obstruction in late phase symptoms.

Alternative therapies such as aromatherapy have become more commonly used in a wide range of health problems including allergic rhinitis patients. Aromatherapy uses purified oils from fragrant plants to help relieve health problems and improve quality of life in general.

S. Y. Choi and K. Park evaluated the ability of aromatherapy using essential oils obtained from sandalwood,* Geranium*, and* Ravensara* to alleviate the symptoms perennial allergic rhinitis in adults. They demonstrated the aromatherapy improved total nasal symptom score, especially in nasal obstruction improving quality of life and fatigue in patients with perennial allergic rhinitis.

Smooth muscle cells play a significant role in the pathogenesis of asthma and are associated with bronchoconstriction, bronchial hyperresponsiveness, inflammation, and airway remodeling.* Cortex phellodendri*, an herb referred to as Huang Bai, is used in traditional Chinese medicines to treat dysentery, jaundice, urinary infection, and rheumatoid arthritis. Berberine, an alkaloid, is the major active constituent of* C. phellodendri*. Berberine was also isolated from* Argemone ochroleuca* (Papaveraceae) and demonstrated relaxant effect on guinea-pig tracheal smooth muscle.

Using an allergic inflammation model in mice (induced by ovalbumin) Q.-J. Jiang et al. were able to show that the* n*-butyl alcohol extract isolated from* C. phellodendri* (which does not contain berberine) inhibited high K^+^- and acetylcholine-induced precontraction of airway smooth muscle in tracheal rings from control and asthmatic mice. In addition, similar results were obtained from lung slices taken from these mice. In follow-up experiments using nifedipine (L-voltage-sensitive Ca_2_^+^ channel blocker) and Pyr 3 (an TRPC3 and Orai1 channels blocker) the authors were able to demonstrate that tracheal rings relaxation responses were mediated by L-type Ca_2_^+^, TRPC3, and/or Orai1 channels. The authors suggest that* n*-butyl alcohol extract of* C. phellodendri* could be used in developing new drug for relieving bronchospasm.

Respiratory syncytial virus (RSV) is the most common cause of hospitalization in infants and young children in the world. Currently, there are several potential treatments for RSV infection in development (including vaccines and therapeutic agents). To date, there is no effective treatment for RSV lower respiratory tract infection. L. L. Lin et al. make a review of traditional Chinese medicine as well as bioactive compounds present in aqueous extracts and formulas derived from traditional Chinese medical herbs with potential to be used in the prevention and treatment of RSV.

Acute lung injury (ALI) and acute respiratory distress syndrome (ARDS) are life-threatening syndromes that cause high morbidity and mortality. They can be caused directly by lung diseases such as such as pneumonia or aspiration of gastric contents or indirectly by systemic diseases with sepsis, burns, and pancreatitis serving as examples. There is a great therapeutic need for agents that have a clear benefit in ALI/ARDS treatment. ALI is characterized by alveolar edema and uncontrolled neutrophil migration to the lung. Unwanted neutrophil activation can lead to tissue damage through the release of proteases, oxidants, and cationic peptides.

Tanreqing injection (TRQ), a water-soluble natural extract from five traditional Chinese medicines,* Scutellariae baicalensis*,* Fel selenarcti*,* Cornu naemorhedi*,* Flos lonicerae*, and* Forsythiae fructus*, has demonstrated antibacterial, antiviral, and anti-inflammatory effects. W. Liu et al. using an acute lung injury model in rats induced by LPS demonstrated that TRQ, administrated by intraperitoneal injection, decreased airways inflammation, mucus production, and proinflammatory cytokines (TNF-*α*, IL-1*β*, IL-6, and IL-8) via regulation of NF-*κ*B, ERK1/2, JNK, and p38 MAPK pathways.

